# Exploring the impact of cancer registry completeness on international cancer survival differences: a simulation study

**DOI:** 10.1038/s41416-020-01196-7

**Published:** 2020-12-09

**Authors:** Therese M.-L. Andersson, Mark J. Rutherford, Tor Åge Myklebust, Bjørn Møller, Isabelle Soerjomataram, Melina Arnold, Freddie Bray, D. Max Parkin, Peter Sasieni, Oliver Bucher, Prithwish De, Gerda Engholm, Anna Gavin, Alana Little, Geoff Porter, Agnihotram V. Ramanakumar, Nathalie Saint-Jacques, Paul M. Walsh, Ryan R. Woods, Paul C. Lambert

**Affiliations:** 1 Department of Medical Epidemiology and Biostatistics, Karolinska Institutet, Stockholm, Sweden; 2 Department of Health Sciences, University of Leicester, Leicester, UK; 3 Cancer Surveillance Section, International Agency for Research on Cancer (IARC/WHO), Lyon, France; 4 Cancer Registry of Norway, Institute of Population-based Cancer Research, Oslo, Norway; 5 Department of Research and Innovation, Møre and Romsdal Hospital Trust, Ålesund, Norway; 6 Nuffield Department of Population Health, University of Oxford, Oxford, UK; 7 King’s College London, Clinical Trials Unit, London, UK; 8 Department of Epidemiology and Cancer Registry, CancerCare Manitoba, Winnipeg, MB Canada; 9 Analytics and Informatics, Ontario Health (Cancer Care Ontario), Toronto, ON Canada; 10 Surveillance and Pharmacoepidemiology, Danish Cancer Society Research Center, Copenhagen, Denmark; 11 Northern Ireland Cancer Registry, Queen’s University Belfast, Northern Ireland, UK; 12 Cancer Institute NSW, Alexandria, NSW Australia; 13 Canadian Partnership Against Cancer, Toronto, ON Canada; 14 Research-Institute, McGill University Health Center, Montreal, QC Canada; 15 Nova Scotia Health Authority Cancer Care Program, Registry & Analytics, Halifax, NS Canada; 16 National Cancer Registry, Ireland Cork, Ireland; 17 Cancer Control Research, BC Cancer, Vancouver, BC Canada

**Keywords:** Epidemiology, Cancer epidemiology

## Abstract

**Background:**

Data from population-based cancer registries are often used to compare cancer survival between countries or regions. The ICBP SURVMARK-2 study is an international partnership aiming to quantify and explore the reasons behind survival differences across high-income countries. However, the magnitude and relevance of differences in cancer survival between countries have been questioned, as it is argued that observed survival variations may be explained, at least in part, by differences in cancer registration practice, completeness and the availability and quality of the respective data sources.

**Methods:**

As part of the ICBP SURVMARK-2 study, we used a simulation approach to better understand how differences in completeness, the characteristics of those missed and inclusion of cases found from death certificates can impact on cancer survival estimates.

**Results:**

Bias in 1- and 5-year net survival estimates for 216 simulated scenarios is presented. Out of the investigated factors, the proportion of cases not registered through sources other than death certificates, had the largest impact on survival estimates.

**Conclusion:**

Our results show that the differences in registration practice between participating countries could in our most extreme scenarios explain only a part of the largest observed differences in cancer survival.

## Background

Population-based cancer registries (PBCR) are a critical component of operational national cancer control programmes. In addition to providing information on current and future requirements for cancer services, they are used to monitor and evaluate prevention, early detection and curative programmes.^1^ Comparisons of cancer survival between registry populations such as those undertaken as part of the International Cancer Benchmarking Partnership (ICBP), CONCORD and EUROCARE^2–4^ have sought to evaluate the effectiveness of cancer services—the efficacy of treatment in the context of how it is applied at the population level—in specific settings. Such studies have undoubtedly been influential in assessing and (re)formulating cancer plans,^5^ and equally have been subject to some concerns regarding their validity due to differences in cancer registration between countries.^6^ Several studies have investigated aspects of registration practice to ascertain whether they can explain observed survival differences between countries,^7–10^ finding that particular registration differences are unlikely to impact greatly on survival differences. Some of these studies use real cancer registry data, and change the data to mimic different potential scenarios in terms of proportion of missing cases or wrong date of diagnosis.^8–10^ However, it is difficult to know if the effect of the changes applied to the data would have the same impact in other population(s), where the data is collected differently. To get a better understanding of the impact of different registration practice or registration problems, simulation studies can be used. The advantage to using simulated data is that the truth is known, and any simulated registration process or error can be compared to the truth or other scenarios. Most potential issues regarding the comparability of cancer patient survival between different registries relate to unknown quantities, such as missing cases or missing information. This can never be fully adjusted for, since if these cases or the information was known, they would be included. Therefore, a simulation approach, where we can create a perfect registry with all cases included, is an easier approach to understand how registration differences impact survival estimates. Rutherford et al.^7^ used a simulation approach to investigate the impact of incomplete registration, but assumed that the probability of registration was not associated with prognosis. This is not realistic if cases with a more severe disease are less likely to be registered. In addition, as all simulations studies, Rutherford et al. only investigated a set of scenarios, and more scenarios could be of interest. Further work is therefore needed to understand each aspect of registry practice and where possible, quantify how it affects cancer survival, according to a range of realistic scenarios.

These issues are complex; for example, two PBCRs could have the same level of completeness of registration but prognosis-related differences in the type of cancer patients more likely to be missed, thus affecting survival estimates. The ICBP SURVMARK-2 study is an international partnership aiming to examine the reasons behind disparities in cancer survival across high-income countries which have high-quality population-based cancer registries. It includes 21 population-based cancer registries covering 21 jurisdictions in seven countries: Australia (New South Wales, Victoria, and Western Australia), Canada (Alberta, British Columbia, Manitoba, New Brunswick, Newfoundland and Labrador, Nova Scotia, Ontario, Prince Edward Island, Quebec, and Saskatchewan), Denmark, Ireland, New Zealand, Norway, and the UK (England, Scotland, Wales, and Northern Ireland). As part of the ICBP SURVMARK-2, we used simulation to assess the impact on survival estimates of differences in registration completeness, of the characteristics of those missed, and of the inclusion of cases found from death certificates. A key objective was to ascertain the specific circumstances when variations in registry practice lead to consequential (or inconsequential) survival differences in benchmarking survival studies for high-income countries.

## Methods

### Definitions: DCN, DCI and DCO cases

**Fig. 1:**
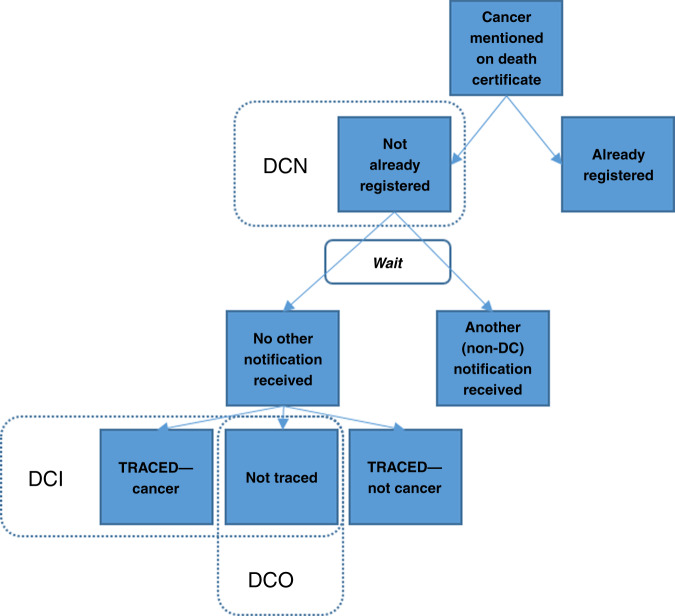
The process of inclusion of cancer cases notified from death certificates in cancer registries. DCN death certificate notified, DCI death certificate initiated, DCO  death certificate only.

Figure 1 gives a graphical representation of the death certificate clearance process described below, and the definition of death certificate notified (DCN), death certificate initiated (DCI) and death certificate only (DCO) cases. PBCRs seek information from the multiple sources in which cancer cases are diagnosed or treated, including hospital records and diagnostic departments, with death certificates providing an important source where cancer is mentioned either as a main or contributory cause of death.^1^ Most of the latter cases will have already been registered from another source; the subset of cases not previously registered are referred to as DCN cases. It is recommended that PBCRs identify a suitable interval to see if another notification arrives for these cases from routine sources.^11–13^ Those for which no other notifications have been received after this interval are then subject to a trace-back enquiry to see if other sources (e.g. clinical or pathology sources) could be found, thus potentially enabling additional information to be retrieved on the case, including the date of incidence. The cases that are deemed reportable are referred to as DCI cases; these are cases that would not have been known by the registry if it was not for the death certificate. For a subset of the DCI cases, there may be no information on date of incidence found through trace-back, and these cases are referred to as DCO cases, and are normally, as a rule, not included in survival estimation.

**Fig. 2:**
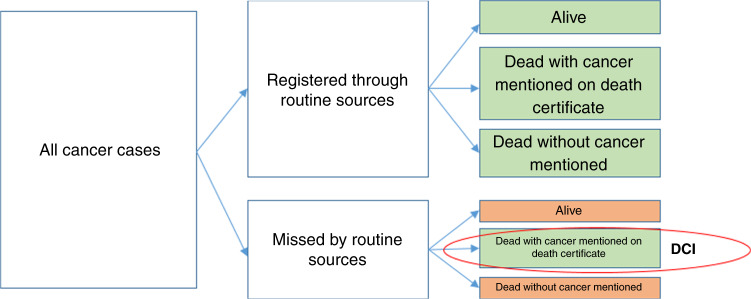
Illustration of Death Certificate Initiated (DCI) cases as a subset of all cases of cancer arising in the population. The left-hand box illustrates all cancer cases in the population, of which some are notified to the registry from sources other than death certificates and some are not. All of the notified cases, together with the cases found through death certificates, the DCI cases, are included in the register (indicated by green boxes). Among the non-notified, cases still alive or who died without cancer mentioned on the death certificate will not be included in the register.

If all cancer cases were notified to the cancer registry when diagnosed, the death certificate information would be inconsequential as a source for adding cases to the register. The inclusion of DCI cases, therefore, reveals that cases are missed by the registry. Among those that are missed, only those who die with cancer mentioned on the death certificate will be included in the registry. These are not a random sample of cases missed and will generally have a relatively worse prognosis since they died due to cancer. Patients who are still alive, or who died without cancer being mentioned on the death certificate, will not be captured. This is illustrated in Fig. 2, where the green boxes represent cases who are included in the cancer register, and the orange boxes are those not included. The inclusion of DCI cases is important when estimating incidence since it increases completeness. However, inclusion of DCI cases when estimating survival, could render biased results. The extent of this bias depends, among other things, on the proportion of missed cases and how these cases compare to the registered cases with respect to their likely prognosis.

### Simulation study

To assess the impact of missed cancer cases in cancer registries on survival estimates we performed a simulation study, that extends the simulation process used by Rutherford et al.^7^ One difference to the simulations by Rutherford et al was to let the probability of not being registered to depend on a prognostic covariate instead of assumed to be random and we also investigated other scenarios. By using simulated data, we know the truth, and can therefore assess the bias in survival estimates introduced by different registration errors. In real data, we don’t know the exact process behind cancer cases being missed. Some of these cases will later be included as DCI cases, however, they are possibly only a subset of the missed cases. To fully understand the impact of missed cases it is therefore not possible to use real data. Using a simulation approach mimicking the registration process is therefore important to understand the impact of missed cancer cases, and the trace-back procedure, on survival estimates.

**Fig. 3:**
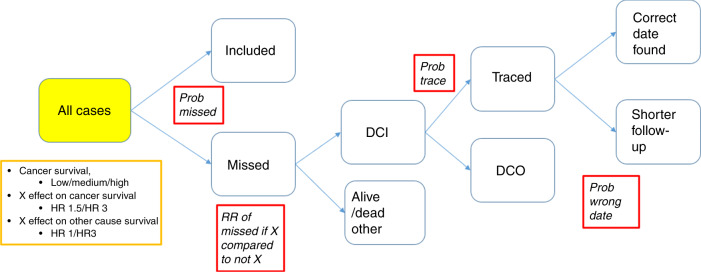
Illustration of the simulation process, the yellow box describes features modified to create the 9 main scenarios and red boxes demonstrate registration errors that were simulated at different levels. HR hazard ratio, Prob probability, RR relative risk, DCI death certificate initiated, DCO death certificate only. X is a prognostic factor present in 25% of the cases.

Firstly, we simulated cancer cases, their age at diagnosis and time to death due to cancer.^14^ Age was normally distributed with a mean of 70 years and a standard deviation of 15 years. We also introduced a prognostic factor, which we denote herein as Factor X, and impose that the time to death due to cancer was dependent on both Factor X and age at diagnosis. The effect of age on cancer mortality was simulated with a time-dependent effect so that the effect of age on the cancer mortality rate decreased with time since diagnosis. The probability of having Factor X was set to 0.25, to create a prognostic factor that is common among the cancer cases but with a majority of cases not having this factor. Time to death due to other causes was also simulated, with the possibility of being dependent on both age and Factor X. For each individual, their cause of death was determined by the minimum survival time out of the two simulated survival times. This gave rise to a dataset that can be regarded as the cancer cases who should be reported to a cancer registry, as seen in the solid yellow box in Fig. 3 and are the cases for which we want to estimate survival. Formulas describing the models used for simulating the cancer-specific and other cause mortality are included in the Supplementary material.

We simulated nine main scenarios, denoted scenario A to I, with 5000 cancer cases in each simulated dataset, and 100 datasets for each scenario. We simulated three levels of cancer-specific (net) survival, low (with a 5-year net survival approximately 21%), intermediate (with a 5-year net survival approximately 36%) and high (with a 5-year net survival approximately 70%), representing a cancer site with poor, moderate or good prognosis, respectively. The hazard ratio (HR) of the prognostic Factor X on the cancer-specific mortality was set to 1.5 or 3, and in scenarios A–F the prognostic factor did not have an effect on other cause mortality (HR = 1). This represents scenarios where the prognostic variable is disease related, e.g. being diagnosed with advanced stage disease. In scenarios G–I, the prognostic factor has an effect on other-cause mortality, with a HR of 3, as well as cancer-related mortality. This represents scenarios where the prognostic factor is not disease-specific, for example, the effect of comorbidity. The scenarios are listed in Table 1.

**Table 1:** Description of the 9 simulated scenarios.

Scenario	Cancer survival	HR for Factor X on cancer-specific mortality	HR for Factor X on other-cause mortality
A	Low	1.5	1
B	Intermediate	1.5	1
C	High	1.5	1
D	Low	3	1
E	Intermediate	3	1
F	High	3	1
G	Low	1.5	3
H	Intermediate	1.5	3
I	High	1.5	3

*HR* hazard ratio.

The next step was to introduce registration errors, and we introduced four factors that can vary in the simulated scenarios, denoted by red boxes in Fig. 3. Firstly, a proportion of cases are not notified to the registry when diagnosed, and the probability that a cancer case is not notified is the first factor in the simulation of registration errors (*Prob misse*d). The probability of not being notified could be different for cases with a worse prognosis (those with Factor X), so there is a relative risk (RR) of being missed for those with Factor X compared to those without Factor X. This RR is the second factor of interest in our simulations (*RR of missed if X compared to not X*). Among the missed cases, those who die due to cancer in our simulated data will be picked up as DCI cases. The probability that a date of incidence is found for a DCI case can vary between registries, and this is the third factor of interest in our simulations (*Prob trace*). The cases where a date of incidence is not found are DCO cases. For those where a date of incidence is found, this might not be the true date, but rather a later date or a recurrence date, so that the probability of having a shorter survival time due to trace-back is the fourth factor taken into account in our simulations (*Prob wrong date*). The simulation process is illustrated in Fig. 3.

For each of the nine main simulated scenarios, 24 different registration errors were simulated, based on the four factors described above. We simulated three probabilities for a case to be missed (0.05, 0.1 and 0.2), two RRs of being missed for those with Factor X compared to those without Factor X (1.5 and 5), two levels of probability of successful trace-back (0.7 and 0.9) for DCI cases, and lastly two probabilities of having a shorter (incorrect) survival time determined following trace-back (0 and 0.3). The true values of these parameters in the ICBP registries are by definition unknown, but we believe them to cover scenarios that are both plausible as well as more extreme.

Within each of the simulated datasets, we estimated the age-standardised 1- and 5-year net survival,^15^ overall and by Factor X, both before and after introducing registration errors. We refer to the absolute difference between the estimates from the dataset prior to (including all cancer cases), and after introducing the registration errors, as the bias introduced by the registration error. The bias is therefore measured as a percentage point difference in survival, and a positive value of the bias indicates an underestimate of survival. Due to the many results only results from scenarios A–C are presented in the main paper and results from scenarios D–I are presented in the Supplementary material.

## Results

**Fig. 4:**
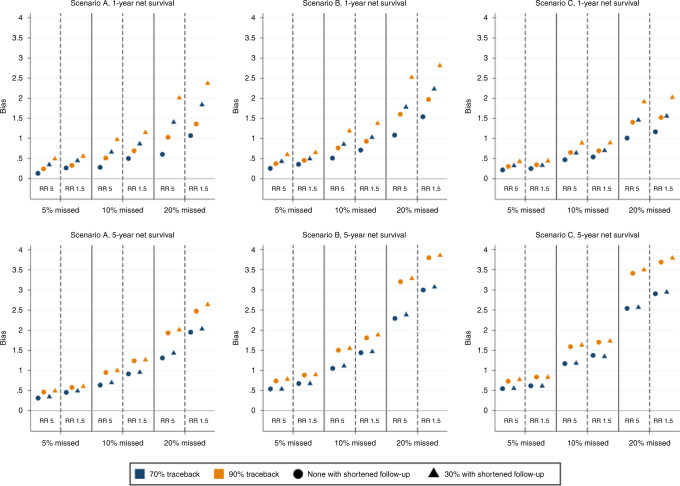
Bias in 1- (upper panel) and 5-year (lower panel) net survival estimates for different simulated scenarios of registration errors, with 5%, 10% or 20% of cases missed at diagnosis, and 70% or 90% of those missed found through trace-back (indicated by blue and yellow markers, respectively). The relative risk (RR) of being missed for those with the prognostic Factor X compared to those without Factor X is 5 or 1.5, and scenarios with unaltered follow-up time (circles) and shortened follow-up time for 30% of those found through trace-back (triangles) are presented. Bias is measured as a percentage point difference, and all registration errors lead to an underestimation of survival.

Figure 4 shows the bias—i.e. the degree to which survival is underestimated (expressed in absolute percentage point differences)—in age-standardised 1- and 5-year net survival for scenarios A–C. In the figure it can be seen that the bias increases as the proportion of missed cases increases. The degree of missingness causes the greatest bias and thus has potentially the greatest impact on comparing net survival, among the four factors assessed in the simulation. Further, it can be seen that a smaller relative difference (e.g., RR of 1.5 versus 5) in the risk of being missed between those with the Factor X compared to those without Factor X leads to a larger bias. A higher probability of successful trace-back (0.9 versus 0.7) also results in a larger bias, as does an incorrect date of diagnosis found by trace-back. The bias in both 1- and 5-year survival is largest for cancers with intermediate survival, but cancers with higher survival have a smaller bias in 1-year survival than cancers with lower survival, while the converse is observed for 5-year survival. The largest observed bias in Fig. 4 is just under 3 percentage points for 1-year survival slightly less than 4 percentage points for 5-year survival.

**Fig. 5:**
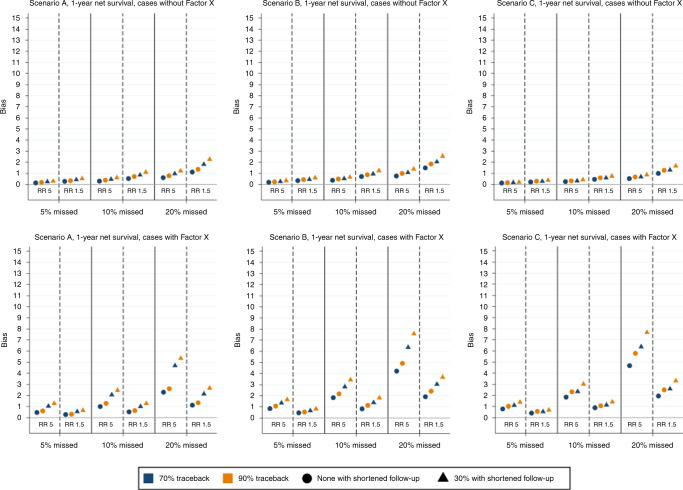
Bias in 1-year net survival estimates for those without (upper panel) and with (lower panel) prognostic Factor X for different simulated scenarios of registration errors, with 5%, 10% or 20% of cases missed at diagnosis, and 70% or 90% of those missed found through trace-back (indicated by blue and yellow markers, respectively). The relative risk (RR) of being missed for those with the prognostic Factor X compared to those without Factor X is 5 or 1.5, and scenarios with unaltered follow-up time (circles) and shortened follow-up time for 30% of those found through trace-back (triangles) are presented. Bias is measured as a percentage point difference, and all registration errors lead to an underestimation of survival.

Figure 5 shows the bias in 1-year age-standardised net survival, separately for those with and without the prognostic Factor X. For those without Factor X, which is approximately 75% of all cancer cases, we see a similar pattern as for all cases overall. However, for those with Factor X the bias is larger, especially with a higher RR of being missed, and the ordering between the three types of cancer prognosis is not the same. In general, the bias is larger for those with Factor X than those without, which is also true for the 5-year age-standardised net survival (Supplementary Fig. S1).

Results from scenarios D–I are presented in the Supplementary material (Figs. S2, S3). The bias is generally larger when the effect of Factor X is stronger (scenarios D-F), but similar or lower for scenarios G–I when Factor X also has an effect on other-cause mortality.

The proportion of DCI and DCO cases for each of the 24 different combinations of registration errors introduced for scenarios A–C are presented in Supplementary Table S1. The proportion of DCIs is driven by the probability of a case failing to be registered from routine sources as well as by the cancer-specific survival. With an increasing probability of being missed and with poorer prognosis, the proportion of DCIs increases. The proportion of DCOs on the other hand is, to a large extent, driven by how likely the trace-back procedure is successful.

## Discussion

The findings from this comprehensive simulation study show that the differences typically seen when comparing cancer survival between the high-income countries included in the ICBP SURVMARK-2 study, are unlikely to be explained by issues relating to completeness of cancer registration. Our simulations show that even in the scenarios when the probability of cases being missed through routine registration sources is as high as 20%, the bias introduced is notably smaller than the largest differences seen between the jurisdictions included in ICBP SURVMARK-2. For the calendar period 2010–2014, the largest difference in age-standardised 5-year net survival was 8.8 percentage points for oesophageal cancer, 12.0 for stomach, 11.9 for colon, 8.7 for rectum, 6.7 for pancreatic, 7.3 for lung and 10.2 for ovarian cancer.^16^ Even so, some of the observed differences in survival might be due to differences in completeness and trace-back procedures. It is, however, important to keep in mind that no PBCR is perfect, so when comparing survival between jurisdictions, all estimates are to some extent affected by differences in registry completeness. All registries within the ICBP SURVMARK-2 include DCI cases, even if they cannot always be distinguished from cases found from other sources, and will therefore tend to underestimate survival. Hence, the differences due to registration practice are probably smaller than the bias, as defined in our simulations, since our comparisons are to a perfect registry.

Cancer registries often report proportions of registrations that are DCOs as a quality indicator. The proportion of DCOs is not an indicator of the completeness of the registry but of the likely validity of the recorded information on the cases in the register. Scenarios with large differences in the probability of missed cases and in the proportions of DCIs can give rise to very similar proportions of DCOs. The true level of completeness is of course difficult to know, if the registry was aware of the missed cases they would be included in the registry. However, the proportion of DCIs is more informative about the percentage missing than the proportion of DCOs. We, therefore, recommend cancer registries report the proportion of DCIs together with the corresponding proportion of DCOs, and to flag and keep track of which cases in the register are DCIs. We acknowledge that such information is not currently routinely recorded, but to better understand issues regarding completeness this information is essential. Methods that use death certificate information for estimating completeness have been suggested, for example, the death certificate and mortality to incidence method and the flow method,^12,17–20^ which provides another compelling reason for cancer registries to record this information.

As with all simulation studies, our study has limitations. Cancer registration is a complex process, and we have had to make some simplifications for our simulations. We have, for example, assumed that cause of death is perfectly recorded. We have investigated a limited number of scenarios, with nine main scenarios and 24 combinations of registration errors, even though more scenarios could be of interest. It is not possible to investigate every single possible scenario on a simulation study. We have selected parameters that represent a range of values that include what we believe to be both plausible and somewhat more extreme scenarios. We have in all scenarios assumed that the prognostic Factor X, that is also associated with the probability of missed registration, is present in 25% of the cases. Changing this proportion could have an impact on our estimates, however, the overall pattern is likely to be the same. We have also limited the purpose of this study to focus on completeness and trace-back, but more work is needed to understand how other aspects of registry practice interact and impact on estimates of cancer survival. Hence as part of the ICBP SURVMARK-2 study, the focus will now turn to investigate the impact of failure of linkage to death information (resulting in ‘immortals’) and issues related to the accuracy of the date of incidence.

Other studies have previously investigated the impact of registration differences on cancer patient survival, and made conclusions similar to ours.^7–10^ Most of the previous studies manipulate real data to mimic what might be occurring in another cancer registry. However, the only way of fully measuring the effect of different registration practice is with the use of simulation studies, where one can create data that represent a perfect registry. Simulation studies can greatly increase the understanding of which factors are of concern when analysing real data. Population-based cancer registries remain an essential source of information, and we believe that our results can be useful when interpreting cancer patient survival differences.

## Disclaimer

Where authors are identified as personnel of the International Agency for Research on Cancer/WHO, the authors alone are responsible for the views expressed in this article and they do not necessarily represent the decisions, policy or views of the International Agency for Research on Cancer/WHO.

## Supplementary information

Supplementary material

## Data Availability

All data used were simulated by the authors. All programs used to create the data can be obtained from the corresponding author upon request.
