# Supplementary material for: Exploring the impact of cancer registry completeness on international cancer survival differences: a simulation study
Source: Br J Cancer. 2020 Dec 9;124(5):1026–32. doi: 10.1038/s41416-020-01196-7 (PMC7921088; doi:10.1038/s41416-020-01196-7)
Supplement: Supplementary file 1 — Supplementary material [file 41416_2020_1196_MOESM1_ESM.pdf]

Supplementary material

The models used for simulating the cancer-specific and other cause mortality.

The Stata program survsim [1] was used to simulate both the time to death due to cancer and time to death due to other causes.

Time to death due to cancer was simulated from a model with the log cumulative hazard expressed as:

$$\ln H(t) = \ln \lambda + \gamma * \ln t + \ln 1.03 * \text{agec} + \ln 0.99 * \text{agec} * \ln t + \ln \text{HRc} * \text{X}'$$

where agec is age at diagnosis centered around the mean value 70, and X' is -0.25 for those without the prognostic Factor X and 0.75 for those with Factor X (e.g. centered around the mean value). HRc is the hazard ratio for the effect of the prognostic Factor X on cancer-specific mortality, and varied across scenarios. The lambda and gamma also varied across the simulated scenarios as seen below.

Level of survival	Scenarios	Lambda	Gamma
Low	A, D, G	0.61	0.63
Intermediate	B, E, H	0.4	0.6
High	C, F, I	0.12	0.64

Age at death due to other causes was simulated from a model with the log hazard expressed as:

$$\ln h(\text{age} + t) = -9.4 + 0.056 * s_1(\text{age} + t) - 0.000046 * s_2(\text{age} + t) + 0.000033 * s_3(\text{age} + t) - 0.000012 * s_4(\text{age} + t) + \ln \text{HRo} * \text{X}'$$

where s(t) are restricted cubic spline functions [2], with knots at the values 18, 38, 58.5, 79 and 99, and age is age at diagnosis. HRo is the hazard ratio for the effect of the prognostic Factor X on other cause mortality, and varied across scenarios.

1. Crowther MJ, Lambert PC. Simulating biologically plausible complex survival data. *Stat Med.* 2013;32(23):4118-4134.
2. Durrleman S, Simon R. Flexible regression-models with cubic-splines. Statistics in Medicine 1989; 8:551–561.

Table S1. The proportion of death certificate initiated (DCI) and death certificate only (DCO) cases in the 3 main simulated scenarios (scenario A-C), for each of the investigated registration errors. Prob = probability, RR = relative risk.

Registration error				Scenario A		Scenario B		Scenario C	
Prob missed	RR missed	Prob trace-back	Prob wrong date	% DCI	% DCO	% DCI	% DCO	% DCI	% DCO
0.05	1.5	0.70	0.0	4.06	1.22	3.28	0.99	1.51	0.46
0.05	1.5	0.90	0.0	4.02	0.41	3.26	0.34	1.47	0.14
0.05	5	0.70	0.0	4.22	1.22	3.45	1.04	1.62	0.50
0.05	5	0.90	0.0	4.21	0.42	3.48	0.35	1.63	0.17
0.05	1.5	0.70	0.30	4.08	1.22	3.27	0.98	1.48	0.44
0.05	1.5	0.90	0.30	4.05	0.40	3.22	0.31	1.52	0.15
0.05	5	0.70	0.30	4.20	1.26	3.50	1.05	1.66	0.50
0.05	5	0.90	0.30	4.19	0.42	3.45	0.34	1.62	0.16
0.10	1.5	0.70	0.0	8.15	2.45	6.67	1.99	3.12	0.94
0.10	1.5	0.90	0.0	8.17	0.82	6.61	0.68	3.13	0.31
0.10	5	0.70	0.0	8.50	2.53	7.02	2.14	3.42	1.02
0.10	5	0.90	0.0	8.46	0.85	7.03	0.70	3.39	0.34
0.10	1.5	0.70	0.30	8.22	2.49	6.62	2.00	3.09	0.95
0.10	1.5	0.90	0.30	8.11	0.82	6.69	0.66	3.07	0.30
0.10	5	0.70	0.30	8.48	2.54	7.05	2.11	3.42	1.02
0.10	5	0.90	0.30	8.40	0.82	7.08	0.70	3.38	0.34
0.20	1.5	0.70	0.0	16.5	4.96	13.8	4.18	6.75	2.02
0.20	1.5	0.90	0.0	16.7	1.70	13.8	1.38	6.66	0.66
0.20	5	0.70	0.0	17.2	5.13	14.6	4.35	7.28	2.19
0.20	5	0.90	0.0	17.2	1.73	14.7	1.49	7.39	0.75
0.20	1.5	0.70	0.30	16.6	4.99	13.8	4.12	6.73	2.01
0.20	1.5	0.90	0.30	16.5	1.65	13.8	1.39	6.72	0.66
0.20	5	0.70	0.30	17.2	5.16	14.5	4.32	7.32	2.18
0.20	5	0.90	0.30	17.2	1.72	14.6	1.46	7.33	0.72

Figure S1. Bias in 5-year net survival estimates for those without (upper panel) and with (lower panel) prognostic Factor X for different simulated scenarios of registration errors, with 5%, 10% or 20% of cases missed at diagnosis, and 70% or 90% of those missed found through trace-back (indicated by blue and yellow markers, respectively). The relative risk (RR) of being missed for those with the prognostic Factor X compared to those without Factor X is 5 or 1.5, and scenarios with unaltered follow-up time (circles) and shortened follow-up time for 30% of those found through trace-back (triangles) are presented.

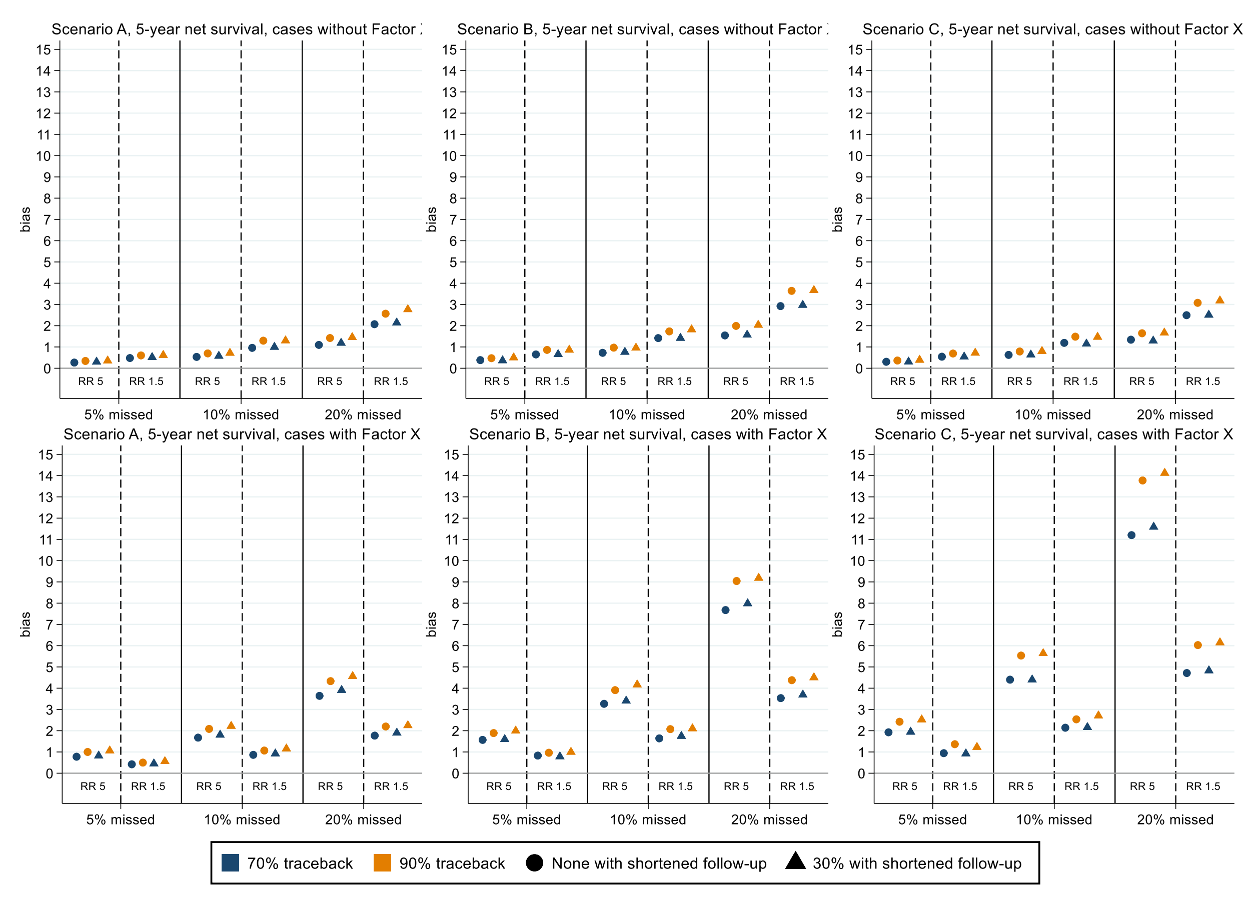

Bias is measured as a percentage point difference, and all registration errors lead to an underestimation of survival.

Figure S2. Bias in 1- (upper panel) and 5-year (lower panel) net survival estimates for different simulated scenarios of registration errors, with 5%, 10% or 20% of cases missed at diagnosis, and 70% or 90% of those missed found through-trace back (indicated by blue and yellow markers, respectively). The relative risk (RR) of being missed for those with the prognostic Factor X compared to those without Factor X is 5 or 1.5, and scenarios with unaltered follow-up time (circles) and shortened follow-up time for 30% of those found through trace-back (triangles) are presented.

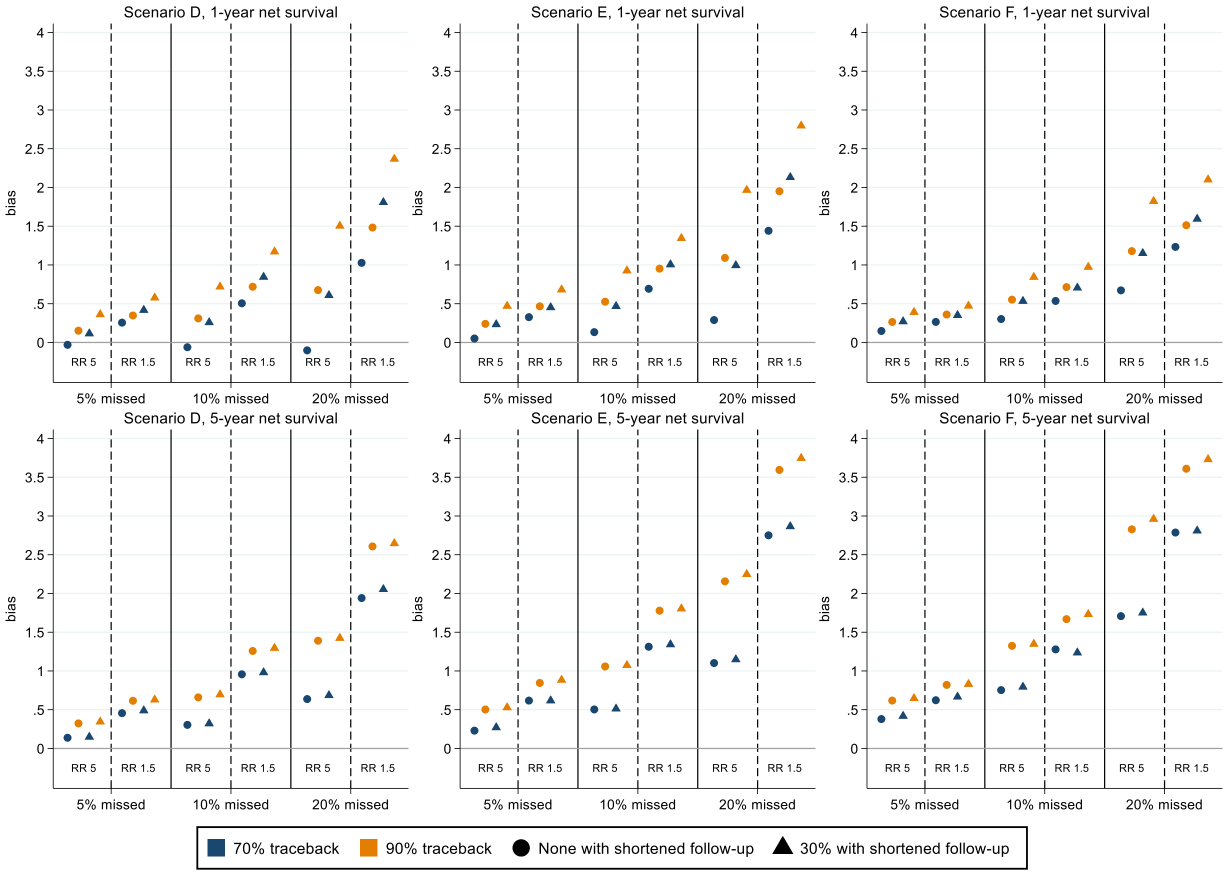

Bias is measured as a percentage point difference, and all registration errors lead to an underestimation of survival.

Figure S3. Bias in 1- (upper panel) and 5-year (lower panel) net survival estimates for different simulated scenarios of registration errors, with 5%, 10% or 20% of cases missed at diagnosis, and 70% or 90% of those missed found through trace-back (indicated by blue and yellow markers, respectively). The relative risk (RR) of being missed for those with the prognostic Factor X compared to those without Factor X is 5 or 1.5, and scenarios with unaltered follow-up time (circles) and shortened follow-up time for 30% of those found through trace-back (triangles) are presented.

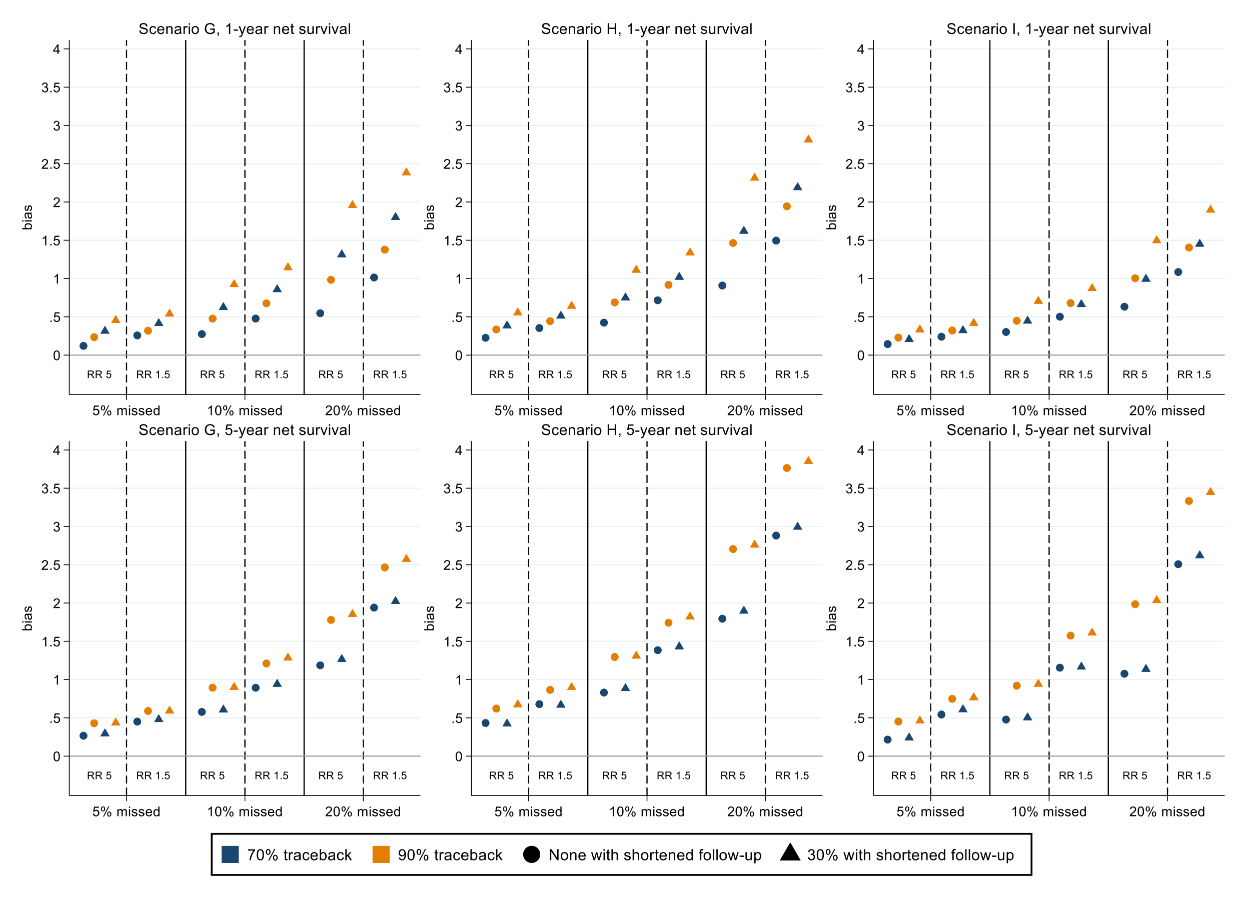

Bias is measured as a percentage point difference, and all registration errors lead to an underestimation of survival.
